# Successful neoadjuvant chemotherapy plus sintilimab for locally advanced cervical cancer: case series and review of the literature

**DOI:** 10.1186/s13000-023-01394-w

**Published:** 2023-09-26

**Authors:** Linlin Liu, Xianbo Deng, Shuang Guo, Shouhua Yang

**Affiliations:** 1 Department of Gynecology and Obstetrics, Union Hospital, Tongji Medical College, Huazhong University of Science and Technology, Wuhan, 430022 Hubei People’s Republic of China; 2 Department of Radiology, Union Hospital, Tongji Medical College, Huazhong University of Science and Technology, Wuhan, 430022 Hubei People’s Republic of China; 3 Department of Pathology, Union Hospital, Tongji Medical College, Huazhong University of Science and Technology, Wuhan, 430022 Hubei People’s Republic of China; 4 Department of Gynecology, South China Hospital, Medical School, Shenzhen University, Shenzhen, 518116 People’s Republic of China

**Keywords:** Locally advanced cervical cancer, Neoadjuvant chemotherapy, Sintilimab, Immune checkpoint inhibitor, Case series

## Abstract

**Background:**

The locally advanced cervical cancer (LACC) of FIGO stage IB3-IIA2 is characterized by large local mass, poor prognosis and survival rate. Tumor response to neoadjuvant chemotherapy for LACC, utilized as a surrogate endpoint, is urgently needed to improve. Given that the antitumor immune response can be suppressed by programed death-1 axis, the treatment paradigm of neoadjuvant chemotherapy combined with immunotherapy has been explored as one of the prognostic treatments in a variety of solid carcinoma. So far, the application of sintilimab, a domestic immune checkpoint inhibitor, combined with neoadjuvant chemotherapy is still limited in LACC, especially in large lesions.

**Case description:**

We present three postmenopausal women diagnosed with FIGO stage IB3-IIA2 cervical squamous cell carcinoma with lesions larger than 5 cm. Demographic, clinical, histopathological, laboratory and imaging data were record. At the completion of the neoadjuvant therapy with paclitaxel plus carboplatin combined with sintilimab, all patients underwent hysterectomy. After neoadjuvant treatment, a pathologic complete response in case 1 and partial responses in case 2 and case 3 were achieved, and neither patient showed any relapse during the follow-up period of 16 to 22 months.

**Conclusions:**

This report provide evidence to support the combination of sintilimab with neoadjuvant chemotherapy in cervical cancer, which has yet to be validated in prospective studies. More clinical data are needed to verify the effectiveness of the combined regimens. This literature review also collected studies involving potential predictors of response to NACT and immunotherapy, which would be helpful in stratifying patients for future trials.

## Introduction

Cervical cancer causes estimated 604,127 new cases and 341,831 mortalities worldwide annually, with a significant proportion of locally advanced cervical cancer (LACC) [1, 2]. As LACC (FIGO IB3-IIA2) is frequently accompanied by large mass, lymph node metastases and easy recurrence [3], with limited intensive treatment options [4], patients usually have a terrible prognosis. Despite a survival benefit has been confirmed under concurrent chemoradiotherapy (CCRT), a 25%-40% recurrence rate [5] and early or late side-effects seriously affect the quality of life [6].

Neoadjuvant chemotherapy (NACT) has emerged as an accepted alternative, which helps downstage tumors before radical surgery, eliminate micrometastases and decrease radiation-related complications [3, 4]. Due to unfavorable outcomes with the delay of curative therapies for NAC refractory LACC patients, current objective response rates (69.4%-90.2%) of NACT needs to be further improved [7], especially in patients with a primary tumor size larger than 5 cm [8]. The discovery that tumor immune evasion promotes NACT chemoresistance via the programed death-1 (PD-1)/PD ligand 1 (PD-L1) inhibitory axis [9, 10] has provided substantial evidence supporting the use of NACT in combination with anti-PD-L1/PD-1 agents for LACC. Sintilimab, a domestically developed PD-1 antibody used in China, has shown similar curative effects, a better safety profile and less financial burden compared to other well-studied immune checkpoint inhibitors (ICIs) such as Nivolumab and Pembrolizumab [11, 12], but a paucity of reports on the successful use of sintilimab combined with NACT in LACC.

In this case report, the patients made a decision for combined treatment using NACT plus sintilimab after in-depth counselling with the gynaecology teams. we hereby present three cases in accordance with the CARE reporting checklist.

## Case presentation

### Case 1

**Fig. 1:**
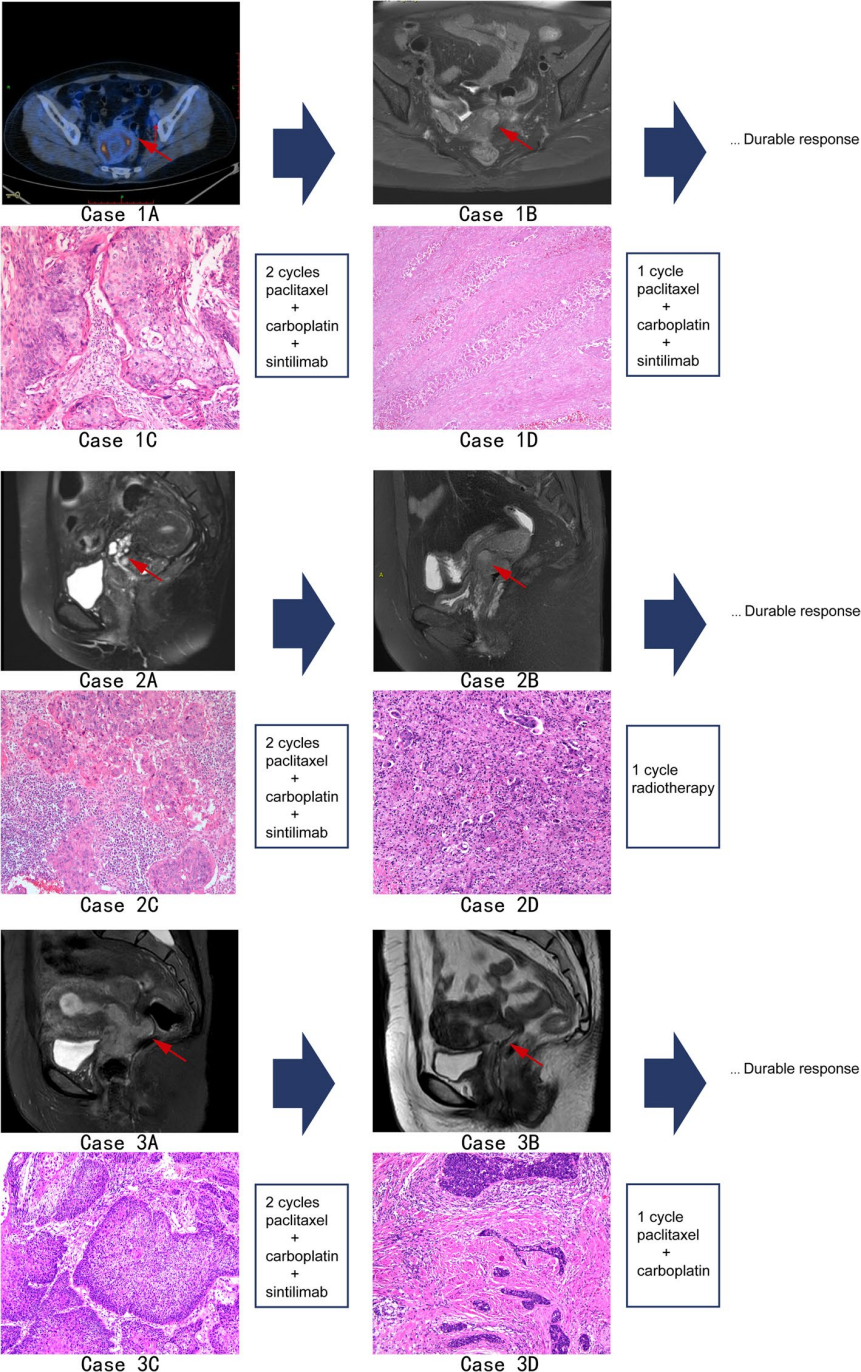
Pathology images and 18F-FDG PET/CT or MRI scans. Notes: Case 1A: 18F-FDG PET/CT image showed a FDG metabolism increasing mass with a maximum diameter of 6.35 cm (thick arrows) and a slightly enlarged lymph node (Size: 0.9*1.0 cm, SUVmax: 1.9) in the left pelvic cavity (thin arrow). Case 1B: T2 sagittal pelvic MRI showed a slight shrinking of the tumor (arrows) after treatment. Case 2A, B: T2 sagittal pelvic MRI scans reflected tumor volumes (arrows) before and after treatment were quantified and compared. Case 3A, B: A decreasing tumor volume assessed by T1 sagittal pelvic MRI (Case 3B) compared with T2 sagittal pelvic MRI image (Case 3A). Case 1-3C: Histopathological biopsy images of hematoxylin and eosin stain from case 1 (× 100), 2 (× 100), and 3 (× 100) showed poorly-differentiated squamous cell carcinoma of cervix. Case 1D: Histopathological images of the surgical specimens (hematoxylin and eosin stain, × 100) revealed a pathological disappearance of all tumor lesions after combined neoadjuvant treatment, compared to pretreatment. Case 2, 3D: Histopathological images of the surgical specimens (hematoxylin and eosin stain, × 100) after combined neoadjuvant treatment showed partial pathological responses in case 2 and 3. 18F-FDG PET/CT, 18fluoro-deoxyglucose positron emission tomography/computed tomography; MRI, magnetic resonance imaging

A 52-year-old Chinese female, who had been menopausal for 4 years, experienced a 5-month history of contact vaginal bleeding and abnormal vaginal discharge accompanied by lower quadrant abdominal pain and distention (Table 1). The patient (gravida 3, para 2, abortion 1) denied any relevant self and family history of cancer or other comorbid medical and surgical events, except for a transabdominal ovarian cystectomy 4 years ago. Whole-body positron emission tomography/computed tomography (PET/CT) was performed, revealing a thickened cervical canal with a measured maximum diameter of 6.35 cm and an increased standard uptake value (SUVmax = 9.2) suggesting malignancy (Fig. 1). Based on the 2018 FIGO system and cervical biopsy specimen findings, the patient was clinically diagnosed with stage IB3 invasive squamous cell carcinoma of the cervix by an expert pathology consultant.

**Table 1:** The clinical characteristics, treatments, and responses of patients in our case

	Case 1	Case 2	Case 3
Age on diagnosis (years)	52	55	53
Pathological diagnosis	SC	SC	SC
Stage	IB3	IIA2	IB3
Primary tumor diameter (maximum, cm)	6.35	5.3	5.9
**Before NACT**			
PD-L1 status	positive	positive	positive
SCC-Ag (ng/ml)	26.9	6.7	8.8
Cytokeratin-19-fragment (ng/ml)	3.93	3.41	6.38
Neutrophil/Lymphocyte	3.54	1.94	2.47
Platelet/Lymphocyte	304.2	131.47	165.33
SIRI = Neutrophil × Monocytes	0.61	0.58	1.78
**Pre-surgery**			
SCC-Ag (ng/ml)	0.6	0.8	1.5
Cytokeratin-19-fragment (ng/ml)	0.76	0.99	1.43
Neutrophil/Lymphocyte	6.36	11.84	1.63
Platelet/Lymphocyte	160.24	90.91	109.27
SIRI = Neutrophil × Monocytes	2.06	5.86	1.16
NACT + ICI (dosage)	Nab-P (260 mg/m^2^), T (AUC6), Sin (200 mg)	P (135 mg/m^2^), T (AUC6), Sin (200 mg)	P (135 mg/m^2^), T (AUC6), Sin (200 mg)
Courses	2	2	2
Response to NACT	pCR	pPR	pPR
Type of surgery	RH + BS + PLND + PALND	RH + BS + PLND + PALND	RH + BS + PLND + PALND
Postoperative treatment	CT + ICI	CRT	CRT
Follow up (mo)	22	16	16
Grade 3–4 adverse events	Myelosuppression (Grade 3)	Leukopenia (Grade 3)	Hypocalcemia, Leukopenia (Grade 3)

*Abbreviations*: *SC* squamous cell carcinoma, *NACT* neoadjuvant chemotherapy, *PD-L1* programed death-ligand 1, *ICI* immune checkpoint inhibitors, *Nab-P* albumin-bound paclitaxel, *P* paclitaxel, *T* carboplatin, *Sin* sintilimab, *pCR* pathological complete response, *pPR* pathological partial response, *RH* radical hysterectomy, *BS* bilateral salpingectomy, *PLND* pelvic lymph node dissection, *PALND* paraaortic lymph node dissection, *CT* chemotherapy, *CRT* chemoradiotherapy

We administered a sequential treatment with two cycles of intravenous PD-1 inhibitor sintilimab (Innovent Biologics, Suzhou, China) 200 mg on day 5 of each 21-day cycle, along with simultaneous NACT consisted of albumin-bound paclitaxel (Shiyao Group Ouyi Pharmaceutical Co., Ltd., Hebei, China) 260 mg/m^2^ on day 1 and carboplatin (Qilu Pharmaceutical Co., Hainan, China) at an AUC6 dose on day 1. After two successive cycles of this selected treatment, the patient subsequently experienced a grade 2 treatment-emergent adverse event of myelosuppression, graded using National Cancer Institute Common Terminology Criteria for Adverse Events (NCI CTCAE) v4.03 criteria. Subsequently, the female patient underwent laparoscopic radical hysterectomy with bilateral salpingectomy and bilateral pelvic lymph node dissection on day 21 after the second cycle of treatment. Based upon the Response Evaluation Criteria in Solid Tumors (RECIST, version 1.1) [13], preoperative MRI (Fig. 1) showed a partial response (44.9% reduction) to treatment. The final pathologic evaluation of surgical specimens (Fig. 1) reported no residual tumor cells in the original tumor bed and with a tumor-free resection margin, indicating a pathological complete response (pCR). All 20 detected lymph nodes were found to be negative. To reduce the recurrence rate after surgery, one cycle of the combination treatment was employed, with no changes in dosage or usage. Following a rapid resolution of symptoms and a remarkable elevated in blood count, the patient was discharged and scheduled for frequent follow-up appointments. At the time of writing, the patient has been disease-free for 22 months.

### Case 2

A previously healthy 55-year-old Chinese female presented with irregular vaginal bleeding for one month after 7 years of menopause (Table 1). Physical examination revealed an exophytic tumor that appeared cauliflower-like. The subsequent cervical biopsy showed an invasive poorly-differentiated squamous cell carcinoma of the cervix. Pelvic MRI (Fig. 1) detected a 5.3 × 3.8 × 3.7 cm cervical mass invading the vaginal wall, which was initially diagnosed as FIGO stage II A2 cervical cancer.

The patient received two cycles of the following neoadjuvant therapies in a 21-day cycle: 135 mg/m^2^ liposomal paclitaxel on day 1, plus carboplatin (AUC 6) on day 1, following by an intravenous infusion of sintilimab 200 mg on day 5. Pelvic MRI (Fig. 1) revealed an overall reduction in the size of the cervical mass (2.6 × 1.9x1.7 cm) compared to the previous MR imaging. After a 3-week rest period following neoadjuvant therapy, the patient underwent laparoscopic radical hysterectomy and bilateral adnexectomy with total pelvic lymph node dissection. Pathological evaluation (Fig. 1) of the surgical specimens revealed a 3.0 × 2.5 cm tumor invading the full thickness of the cervix, with lymphovascular tumor thrombus, no cancer involvement in the surgical margin and partial lymph nodes metastasis (10 out of 24 pelvic lymph nodes), suggesting a pathologic partial response (pPR) to neoadjuvant therapy. Postoperative chemotherapy with pelvic intensity-modulated radiotherapy (46.8 Gy/26 fractions) was administered as a suppletory treatment based on current guidelines in light of high-risk of lymph nodes metastasis [14]. The main treatment-related adverse event was grade 3 leukopenia, evaluated after each cycle of neoadjuvant treatment and postoperative chemotherapy using version 5 of the NCI-CTCAE. Follow-up MRI performed 3 months after the operation showed no evidence of abnormalities. No residual tumor, local recurrence, or remote metastasis was observed during the 16-month follow-up period.

### Case 3

The patient was a 53-year-old post-menopausal woman with well-controlled hypertension, who presented with 1-year intermittent vaginal bleeding (Table 1). The biopsy from a cauliflower-like cervical mass confirmed cervical squamous cell carcinoma. After the follow-up pelvic MRI (Fig. 1) found a 59 × 32 × 41 mm cervical mass without evidence of vaginal wall or pelvic wall involvement, the initial diagnosis was determined as FIGO stage I B3.

The patient was assigned to neoadjuvant therapy comprising two cycles of liposomal paclitaxel (135 mg/m^2^) plus carboplatin (AUC = 6) combined with sintilimab (200 mg on day 5), administered every three weeks. MRI assessment of tumor response showed a reduction in the size of the original cervical lesion to 23 × 18 × 10 mm following treatment (Fig. 1). Laparoscopic radical hysterectomy with bilateral salpingo-oophorectomy, along with total pelvic lymph node dissection, was performed, and a pathological partial response (pPR) was achieved by neoadjuvant treatment (Fig. 1). Due to the involvement of pelvic lymph nodes, the patient received one course of adjuvant chemotherapy (paclitaxel plus carboplatin) as well as brachytherapy at 1 month and 4 months after surgery. The combination therapy was well-tolerated, with most adverse events being grade 1 or 2, whereas grade 3 hypocalcemia and leukopenia occurred. No locoregional recurrences or distant metastasis were observed during the follow-up period of 16 months.

## Discussion and conclusion

These successful cases appear to be the first report involving neoadjuvant sintilimab and chemotherapy for > 5 cm LACC, where the current standard treatment is CCRT. However, due to the financial burdens and patients’ dissatisfaction with the side effects of CCRT, NACT followed by hysterectomy has become a mainstay therapeutic option in some countries in Europe [15] and Asia [16]. Indeed, the presence of an intact tumor vascular bed in untreated tumor tissue allows for the accumulation of chemotherapy drugs in the primary tumor, which helps control the lesion, reduce parametrial infiltration rates, and potentially avoid the complications associated with postoperative radiotherapy that can negatively impact quality of life [17]. Although a single-center phase III randomized controlled clinical trial demonstrated no obviously superiority in 5-year overall survival between NACT and CCRT (75.4% vs. 74.7%, *P* = 0.87) [18], incorporating platinum-based NACT in the therapeutic strategy has been shown to be feasible for LACC, given the favorable prognosis conferred after achieving a pCR with NACT [19]. Since ineffective NACT may worsen the prognosis by delaying the initiation of core therapy [8], current research focuses on improving the efficacy of NACT.

The recent introduction of ICIs targeting PD-1/PD-L1 in frontline treatment has revolutionized the therapeutic management of cervical cancer [20], promoting the antitumor response of T lymphocytes and overcoming the adaptive immunotherapy resistance induced by chemotherapy in cervical cancer [11]. The novel strategy of combining NAC with ICIs, such as pembrolizumab and bevacizumab [11], has shown a synergistic effect in preclinical studies (Table 2). In particular, a large randomized, double-blind clinical trial (NCT03635567) investigating the combination of pembrolizumab (a PD-L1 inhibitor) with NAC in PD-L1-selected patients with persistent, recurrent, or metastatic cervical cancer has revealed superior overall survival (OS) and progression-free survival (PFS) compared to chemotherapy alone. However, there is a lack of sufficiently powered early-phase clinical trials assessing the survival benefit of this combination therapy in patients with LACC.

**Table 2:** Clinical trials of ICIs combination approaches in cervical cancers and sintilimab-based regimens in different tumor types

Drugs	Identifier	Phase	Intervention model	Simple size	Stage	Pathology	ORR^a^(%)(95CI)	mOS(months)(95CI)
**ICIs combined with NACT**								
Pem 200 mg + Platinum + PTX 175 mg/m^2^	NCT03635567	III	double-blind	308	M/R/P	SCC/ACC/ASC	65.9	10.4
BEV 15 mg/kg + Platinum + Taxanes	NCT00803062	III	Single-arm	227	M/R/P	SCC/ACC/ASC	49.3	16.8
BEV 15 mg/kg + Platinum + PTX 175 mg/m^2^	NCT03556839	III	Single-arm	202	M/R/P	SCC/ACC/ASC	NA	NA
Ate 1200 mg + BEV 15 mg/kg + Platinum + PTX 175 mg/m^2^	NCT03556839	III	Single-arm	202	M/R/P	SCC/ACC/ASC	NA	NA
BCD-100 3 mg/kg + Platinum + PTX 175 mg/m^2^	NCT03912415	III	double-blind	158	M/R/P	SCC/ACC/ASC	NA	NA
Tis 200 mg + Platinum + Taxanes	NCT05013268	I	Single-arm	15	LACC	SCC	NA	NA
Sin 200 mg + PTX 150 mg/m^2^ + Cis 70 mg/m^2^	NCT04799639	II	Single-arm	47	LACC	SCC/ACC/ASC	NA	NA
**Sintilimab-based regimens**^b^								
Sin 200 mg + CBP AUC5 + GEM 1000 mg/m^2^	ChiCTR1900023758	II	Single-arm	50	IIIA	NSCLC	46	85.3(NA)
Sin 200 mg + CBP AUC5 + PTX 135 mg/m^2^	ChiCTR1900026593	II	Single-arm	47	II-IVA	ESCC	25.5	14.6(11.3–24.0)
Sin 2 mg/kg + OXA 130 mg/m^2^ + CAP 1000 mg/m^2^	ChiCTR2000030414	II	Single-arm	30	cT3/4aN + M0	GAC	70	13(3.5–19.3)
Sin 200 mg + Platinum + Taxanes	NCC2017A24	II	Single-arm	96	II–IVA	ESCC	62.5	8.9(6.2–14.3)
Sin 200 mg + DTX 75 mg/m^2^	ChiCTR2000030414	II	Single-arm	30	III-IV	NSCLC	36.7	13.4(6.37–20.43)

*Abbreviations*: *ORR* objective response rate, *mOS* median overall survival, *Pem* pembrolizumab, *M/R/P* metastatic/recurrent/persistent, *SCC* squamous cervical carcinoma, *ACC* adenocarcinoma of the cervix, *ASC* adenosquamous carcinoma of the cervix, *BEV* bevacizumab, *PTX* paclitaxel, *NA* not available, *Ate* atezolizumab, *Tis* tislelizumab, *Sin* Sintilimab, *CBP* carboplatin, *GEM* gemcitabine, *NSCLC* non-small-cell lung cancer, *OXA* oxaliplatin, *CAP* capecitabine, *GAC* gastric adenocarcinoma, *ESCC* esophageal squamous cell carcinoma, *DTX* docetaxel

*Notes*: ^a^ORR defined as complete response and partial response according to RECIST 1.1

^b^Clinical trials of combination of sintilimab with different chemotherapy drugs in various types of cancers

Sintilimab is a novel, safe, and effective human monoclonal antibody that blocks the interaction between PD-1 and PD-L1 or PD-L2 [21]. It has the potential to have a greater affinity than pembrolizumab [22]. The safety and efficacy of combining sintilimab with different regimens have been clinically demonstrated in various types of tumors, and preliminary data have showed a significant antitumor effect of this agent (Table 2). Ongoing non-randomized early-phase clinical trials (NCT04799639) (Table 2) are currently underway to evaluate the short-term efficacy and long-term outcomes of combining cisplatin-based NACT with sintilimab in LACC, but the efficacy of these combination regimens has not been definitively proven. In this case series, three patients diagnosed with LACC received three cycles of a triple-drug preoperative regimen consisting of albumin-bound/liposomal paclitaxel, carboplatin, and sintilimab. They achieved objective response, including one complete response and two partial responses, with PFS of 22, 16, and 16 months, respectively. It should be noted that the independent role of albumin-bound paclitaxel in achieving the observed complete response in case 1 cannot be completely ruled out, considering that the recommended therapeutic dose of nab-paclitaxel (260 mg/m^2^) is nearly twice that of liposomal paclitaxel (135 mg/m^2^). Nab-paclitaxel has been recommended as a second-line treatment option for recurrent or metastatic cervical cancers according to the NCCN guidelines [4], and a retrospective study showed slightly increased efficacy (92.3% vs. 82.1%, *P* = 0.042) without increased toxicity [23]. However, due to economic barriers, the two patients in case 2 and case 3 received conventional paclitaxel, which has been widely used for many years.

### Selection of patients suitable for NACT

In clinical settings, there is a requirement to select candidates eligible for NAC, due to the potential risk of cancer progression in chemo-resist patients during the course of NAC treatment. An effective response to chemotherapy is often considered a positive predictor for improved long-term prognosis in LACC patients [24]. To identify potential predictive markers for NACT response, we have summarized relevant literature published over the past 10 years (Table 3). Data from retrospective studies [8, 25–27] suggest that clinical factors, such as tumor size and age at diagnosis, may affect the treatment effects of NAC. However, the clinical features vary greatly among the populations studied, making it difficult to estimate the appropriate values. Additionally, numerous studies have explored whether abnormal expression of certain molecular markers is associated with tumor resistance to chemotherapy, such as VEGF, HIF-1a, EGFL7, and LDH [28–31]. These markers have been linked to hypoxia and angiogenesis in the tumor microenvironment and may be related to drug concentrations in neoangiogenesis regions. Therefore, they could potentially serve as markers for predicting short-term NAC response in the future.

**Table 3:** Potential biomarkers for NACT short-term response prediction in cervical cancer

Biomarkers	Country	Study	Tumor	No. patients	Therapy strategy	CR + PR Rate	*P*-value^a^
			(Stage)			or Median	
**Biomarkers of neoadjuvant chemotherapy**							
**Primary tumor size (cm)**							
> 5 (vs. 4–5) [26]	China	Prospective	CC	157	Cis + VCR + BLM + Surg	72% (vs. 88%)	0.018
			IB/IIA				
≥ 5 (vs. < 5) [8]	China	Retrospective	CC	219	PTX + CBP + Surg	62% (vs. 56%)	0.027
			IB2/IIA2				
> 8 (vs. ≤ 8) [25]	China	RCT	CC	72	Cis + MC + 5-FU + Surg	42% (vs. 75%)	0.029
			IB2/IIB				
**Age (years)**							
≥ 35 (vs. < 35) [26]	China	Prospective	CC	157	Cis + VCR + BLM + Surg	83% (vs. 53%)	0.029
			IB/IIA				
≥ 35 (vs. < 35) [27]	China	Retrospective	SCC	851	Platinum-based + Surg	81% (vs. 88%)	0.041
			IB1/IIB				
**Blood biomarkers**							
NLR	Rome	Prospective	CC	37	Cis + PTX + Surg	2.8 (vs. 4.41)	0.032
(response vs. nonresponse) [9]			IB2/IVA				
PLR	Rome	Prospective	CC	37	Cis + PTX + Surg	1.48 × 10^5^ (vs. 1.78 × 10^5^)	0.026
(response vs. nonresponse) [9]			IB2/IVA				
SIRI	China	Retrospective	CC	187	Cis + PTX + Surg	0.75 vs. 1.29	0.001
(response vs. nonresponse) [32]			IB2/IIA2				
SCC-Ag > 3.5 ng/mL (vs. ≤ 3.5) [33]	China	Retrospective	CC	286	Cis + PTX/Cis + CPT	66% (vs. 82%)	0.010
			IB1/IIIB				
**Other biomarkers**							
HIF-1α ≥ 6^b^ (vs. ≤ 4^b^) [29]	China	Retrospective	CC	59	Cis + PTX	75% (vs. 95%)	0.025
			IIB/IIIB				
PRMT1 ≥ 6^b^ (vs. ≤ 4^b^) [34]	Japan	Retrospective	CC	53	Cis + PTX	40% (vs. 69.6%)	0.033
			IIIB				
Galectin-1	China	Prospective	CC	35	Cis + 5-FU + MC	8.0 vs. 12.0	0.020
(response vs. nonresponse) [35]			IB2-IIA2				
Integrin α5β1	China	Prospective	CC	35	Cis + 5-FU + MC	6.0 vs. 8.0	0.005
(response vs. nonresponse) [35]			IB2-IIA2				
XPA ≥ 4^b^ (vs. ≤ 3^b^) [36]	Japan	Retrospective	CC	56	Cis + PTX	41% (vs. 88%)	0.001
			IIIA-IIIB				
UCP2 ≥ 8^b^ (vs. ≤ 6^b^) [37]	Japan	Retrospective	CC	58	Cis + PTX	49% (vs. 76%)	0.041
			IIIA-IIIB				
TBX2 ≥ 6^b^ (vs. ≤ 4^b^) [38]	Japan	Retrospective	CC	46	Cis + PTX	36% (vs. 76%)	0.009
			IIIA-IIIB				
EGFL7 ≥ 8^b^ (vs. ≤ 6^b^) [28]	Japan	Retrospective	CC	63	Cis + PTX	19% (vs. 86%)	0.001
			IIIA-IIIB				
**Biomarkers of immunotherapy**							
TMB-high (vs. TMB-low) [39]	China	Retrospective	CC	32	Cam + Apa	83% (vs. 43%)	< 0.050
			M/R/P				
PD-L1 ≥ 1% (vs. < 1%) [40]	America	Phase I trial	Solid tumor	132	Keytruda	22% (vs. 4%)	0.021
MMRd/MSI-H (vs. MMRp/MSS) [41]	Korea	Retrospective	Gynecologic cancers	1093	Keytruda + OPDIVO	29% (vs. 12%)	< 0.050

*Abbreviations*: *CR* complete response, *PR* partial response, *CC* cervical carcinoma, *Cis* cisplatin, *VCR* vincristine, *BLM* bleomycin, *Surg* surgery, *SCC* squamous cervical carcinoma, *PTX* paclitaxel, *CBP* carboplatin, *MC* mitomycin C, *5-FU* 5-fluorouracil, *NLR* neutrophil to lymphocyte ratio, *PLR* platelet to lymphocyte ratio, *SIRI* systemic inflammatory response index, *CPT* irinotecan, *HIF-1α* hypoxia inducible factor-1alpha, *PRMT1* protein arginine methyltransferase, *XPA* xeroderma pigmentosum complementation group A, *UCP2* uncoupling protein 2, *TBX2* T-box 2, *EGFL7* epidermal growth factor-like domain 7, *TMB* tumor mutational burden, *M/R/P* metastatic/recurrent/persistent, *Cam* camrelizumab, *Apa* apatinib, *PD-L1* programed death-ligand 1, *Keytruda* pembrolizumab, *MMRd/MSI-H* mismatch repair deficiency/high microsatellite instability, *MMRp/MSS* mismatch repair proficiency/microsatellite stable, *OPDIVO* nivolumab

*Notes*: ^a^χ2 test; ^b^The expression levels of each biomarker were assessed quantitatively by immunohistochemical staining, using a weighted score method as follows: 0, ≤ 5%; 1, 5% to 25%; 2, 25% to 50%; 3, 50% to 75%; 4, > 75%

Recent retrospective and prospective studies have evaluated peripheral blood markers as potential biomarkers of complete response to NACT. These markers include pretreatment serum squamous cell carcinoma antigen (SCC-Ag) levels ≥ 5 ng/ml, low neutrophil/lymphocyte ratio (NLR), and platelet/lymphocyte ratio (PLR) (Table 3) [9, 17]. Inflammation plays a significant role in neoplastic progression. Menter et al. suggested that activated platelets promote tumor growth by releasing vascular endothelial growth factor (VEGF). Elevated levels of inflammatory neutrophil can also affect the sensitivity of tumor cells to chemotherapy by releasing inflammatory mediators and angiogenesis-related VEGF. Previous studies have identified posttreatment levels of SCC-Ag (> 3.5 ng/mL vs. ≤ 3.5 ng/mL: 66% vs. 82%) [33], NLR (response vs. nonresponse: 2.80 vs. 4.41) [9], PLR (response vs. nonresponse: 1.48 × 10^5^ vs. 1.78 × 10^5^) [9], and SIR (response vs. nonresponse: 0.75 vs. 1.29) [32] as independent prognostic indicators. In the present case, all three patients showed a decrease of more than 80% in SCC-Ag levels and more than 30% in PLR relative to the baseline value after receiving neoadjuvant chemoimmunotherapy. Only case 3 displayed a decrease in NLR and SIR. These changes in biomarker levels mirrored a potential benefit from the preoperative combined treatment. Other biomarkers listed in Table 3 provide guidance to clinicians in selecting suitable patients for NACT.

Radical hysterectomy is considered a valid treatment choice after NACT in LACC as started in the guidelines. Postoperative pathological risk factors, such as large tumor size, lymphovascular space invasion, and deep stromal invasion, can help guide adjuvant strategies and assess prognosis [14]. To optimize the accuracy of predicting long-term prognosis, machine learning models have recently emerged as promising techniques. These models integrate clinical parameters, radiomics features and pathology to account for the significant heterogeneity of factors influencing OS and DFS outcomes. Recent studies have explored the impact of factors such as large tumor size, high tumor grading, lymph nodal involvement, parametrial involvement as independent prognostic risk factors in LACC patients who received NACT followed by radical hysterectomy [42, 43] and also developed robust nomograms to predict treatment outcomes [43], which represents a promising direction for future research.

### Combination of NACT with ICIs

Several tissue-based biomarkers have shown a predictive role in immunotherapy efficacy, including (but not limited to) tumor mutation burden (TMB) > 5 mutations/Mb [39], microsatellite instability high (MSI-H) [41], and positive PD-L1 expression (Table 3) [40]. Moreover, recent clinical trials suggest that PD-L1 immunohistochemistry may serve as a marker for predicting the efficacy of combined NACT plus ICI in non-small cell lung cancer [44]. However, these findings are inconsistent with conclusions drawn in locally advanced esophageal squamous cell carcinoma [45]. Further research is needed to validate whether PD-L1 or other molecules could serve as promising markers to stratify LACC patients for appropriate therapies.

The major limitations of our study include the absence of a control or comparison group and a limited sample size. Nonetheless, our study offers a potential opportunity for NACT plus sintilimab in the treatment of LACC. Further clinical trials are necessary to determine the clinical utility of this novel approach for LACC.

## Conclusions

Our case series demonstrated that LACC patients treated with platinum-containing NACT plus PD-L1 inhibitor Sintilimab, followed by hysterectomy, achieved favorable clinical responses with an excellent safety profile. Grade 3 myelosuppression were the main drug-related adverse event observed. In addition, we conducted a systematic review to identify previously published markers for NACT and immunotherapy and found potential utility in blood based inflammatory markers and PD-L1 status as biomarkers.

## Data Availability

The original contributions presented in the study are included in the study and supplementary material.
